# Characterization of an endoplasmic reticulum stress-associated lncRNA prognostic signature and the tumor-suppressive role of RP11-295G20.2 knockdown in lung adenocarcinoma

**DOI:** 10.1038/s41598-024-62836-z

**Published:** 2024-05-29

**Authors:** Liying Yu, Shuang Zhou, Wencong Hong, Na Lin, Qingshui Wang, Pingping Liang

**Affiliations:** 1 Central Laboratory, The Second Affiliated Hospital of Fujian Medical University, Quanzhou, 362000 China; 2 Second Affiliated Hospital of Fujian Medical University, Quanzhou, 362000 China; 3 Pathology Department, The Second Affiliated Hospital of Fujian Medical University, Quanzhou, 362000 China; 4 Fujian-Macao Science and Technology Cooperation Base of Traditional Chinese Medicine-Oriented Chronic Disease Prevention and Treatment, Innovation and Transformation Center, Fujian University of Traditional Chinese Medicine, Fuzhou, 350001 China; 5 Center for Infection and Immunity, Guangdong Provincial Engineering Research Center of Molecular Imaging, The Fifth Affiliated Hospital of Sun Yat-sen University, Zhuhai, 519000 China

**Keywords:** Lung adenocarcinoma, Endoplasmic reticulum stress, lncRNA, RP11-295G20.2, Prognosis, Immunity, Gene ontology, Gene regulation

## Abstract

Endoplasmic reticulum stress (ERS) is commonly induced by accumulating misfolded or unfolded proteins in tumor microenvironment. Long non-coding RNAs (lncRNAs) play important roles in ERS response and lung adenocarcinoma (LUAD) progression. However, the role of ERS-related lncRNAs in LUAD remains unknown. In this study, we aimed to identify ERS-associated lncRNAs with prognostic value in LUAD and characterize their clinical implications. Cox and least absolute shrinkage and selection operator regression analyses identified nine ERS-related lncRNAs with independent prognostic abilities, including five protective factors (CROCCP2, KIAA0125, LINC0996, RPARP-AS1 and TBX5-AS1) and four risk factors (LINC0857, LINC116, RP11-21L23.2 and RP11-295G20.2). We developed an ERS-related lncRNA risk prediction model in predicting overall survival of LUAD patients, which classified TCGA cohorts into high-risk (HS) and low-risk (LS) groups. Comprehensive bioinformatic analyses revealed HS patients featured with late-stage tumors, greater mutation burdens, weaker anti-tumor immunity/responses, and lower sensitivity to targeted drugs compared to LS patients, contributing to tumor progression and a poor prognosis. Functional enrichment analysis implicated these ERS-related lncRNAs in cell migration, cell death, and immunity. Furthermore, expression of the most significantly upregulated risk lncRNA, RP11-295G20.2, was validated at the mRNA level using clinical LUAD samples. Knockdown of RP11-295G20.2 obviously reduced ERS and suppressed proliferation, invasion, and migration of LUAD cells. This novel ERS-related lncRNA signature provides a new biomarker for prognostic prediction, and ERS-associated RP11-295G20.2 serves as a potential therapeutic target in LUAD.

## Introduction

LUAD is the predominant histological subtype of lung cancer and the leading cause of cancer death worldwide, accounting for more than one million deaths per year^1,2^. From 2000 to 2007, the overall 5-year survival rate for lung cancer was 13%^3^. Although patients with stage I lung cancer have a much higher 5-year survival rate (57%) than patients with stage IV (4%)^4^, only 21% of lung cancers are diagnosed at stage I which is one-third of those diagnosed at stage III or IV^4^. Currently, the tumor, node, and metastasis (TNM) staging system is the main prognostic indicator for LUAD. Despite significant progress in gene-targeted therapies and immunotherapies, most patients continue to experience drug tolerance or ineffective drugs due to the high heterogeneity of lung cancer^5^. There remains an urgent need to discover novel biomarkers that can reliably stratify disease aggressiveness and predict patient outcomes in LUAD beyond conventional clinicopathological factors. Thus, early detection of molecular markers and technological advances in genomics and genetics is critical to improving the survival of lung cancer patients.

The endoplasmic reticulum (ER) is a eukaryotic organelle that processes protein handling, modification and folding^6^. Under stress conditions, such as nutrient deficiency, oxidative stress, and drug toxicity, the disrupted protein-folding capacity of the ER causes an accumulation of misfolded or unfolded proteins, resulting in endoplasmic reticulum stress (ERS) state^7^. When a certain level of misfolded proteins is reached, ER molecular chaperones bind to stress receptors including IRE1, PERK and ATF6 on the ER membrane to activate the ERS response^8^. Glucose-regulated protein 78 (GRP78) is a major ER molecular chaperone and has been considered as an ERS biomarker^9^. It is commonly overexpressed in various cancer cells and protects cancer cells from apoptosis^10^. The ERS response has three functional components: activation of genes that increase the functional capacity of the ERS or enhance ER-associated protein degradation; inhibition of translation to reduce client protein flux; and activation of cell death pathways^11^. Apoptosis caused by ERS contributes to the pathophysiology of a variety of diseases, including cardiovascular^12^, neurodegenerative^13^ and metabolic^14^ diseases, and cancer^15^. The persistent ERS in tumor cells is caused by adverse and complex (e.g., ischemia and hypoxia) microenvironments because of enriched diverse genetic, transcriptional and metabolic abnormalities, which ultimately influence their functions, fate and survival^15^. Aberrant activation of ERS sensors and their downstream signaling pathways have thus emerged as key regulators of tumor growth and metastasis, as well as response to chemotherapy, targeted therapies, and immunotherapy.

Long non-coding RNAs (lncRNAs) are transcripts with more than 200 nucleotides that do not encode proteins^16^. They are important in the transcriptional regulation of RNAs and proteins, as well as the organization of nuclear domains^17^. LncRNAs have been implicated in various biological processes, including immune function and tumorigenesis^18,19^. It has been discovered that lncRNAs act as crucial mediators in regulating ERS metabolism in tumorigenesis. ERS-associated lncRNAs (ERS-lncRNAs) can be classified as negative or positive lncRNAs based on associations between lncRNA expression and tumor cell proliferation. Positive ERS-lncRNAs are tumor inhibitors whose activation will inhibit tumor cell proliferation or promote cell death. For example, maternally expressed gene 3 (MEG3), a lncRNA, acts as a tumor suppressor, inhibiting cancer cell proliferation and partially inducing apoptosis via ERS activation^20,21^. The transcriptional suppression of lncRNA GAS5 reduced the ERS-induced osteosarcoma cell apoptosis^22^. The negative ERS-lncRNAs act as tumor facilitators whose activation will increase tumor cell proliferation and decrease tumor cell death. For example, the lncRNA MIAT promotes drug resistance in breast cancer by up-regulating its expression during ERS induced by the effective anticancer drug 5-Fluorouracil (5-FU)^23^. By regulating ERS, lncRNA H19 knockdown inhibits gastric tumor cell migratory ability and proliferation^24^. Overexpression of the lncRNA *HITTERS* increased human oral squamous cell carcinoma cell proliferation, DNA replication, and colony formation by regulating both the DNA damage response (DDR) and the ERS^25^. In LUAD, two studies have identified ERS-related lncRNAs prognostic signatures using the Tumor Cancer Genome Atlas (TCGA) dataset. One signature was based on four increased lncRNAs (KTN1-AS1, CASC15, AC026356.1, and AL606489.1) and one down-regulated lncRNA (MIR223HG) in LUAD tissues^26^. The other signature included other five ERS-lncRNAs (AL606489.1, LINC02178, LINC01117, OGFRP1, and AC087588.1)^27^. However, the mechanism of ERS-lncRNAs is almost unexplored. Exploring clinical implications with more datasets and elucidating molecular mechanisms of ERS-related lncRNAs in LUAD are of great significance that could provide novel insights into LUAD prognosis and therapy.

In this study, we aimed to develop an ERS-related lncRNA signature to independently refine LUAD prognosis prediction and to identify high-risk patient subgroups most likely to benefit from adjuvant therapies. We systematically characterized ERS-related lncRNAs and developed a risk model to predict LUAD prognosis using the TCGA and Gene Expression Omnibus (GEO) datasets. A nine-lncRNAs-based signature was constructed for LUAD patients based on the TCGA dataset and validated using other independent datasets from the GEO. Functional enrichment and immune infiltration analyses were carried out to determine the potential molecular mechanism. Moreover, a risk ERS-related lncRNA with the most significant up-regulation (RP11-295G20.2) in LUAD was selected to reveal its effects on cell ERS, proliferation, invasion and immigration by cell function assays. Our findings provide a novel biomarker and new insights into the molecular basis for predicting immunotherapeutic response and clinical treatment application in LUAD.

## Materials and methods

### Dataset collection

The third-level expression data of RNA-sequencing (RNA-seq) and the corresponding clinical information of LUAD patients (n = 515) were obtained from the TCGA database. Samples with inadequate clinical information and a follow-up period of 0 days were excluded. The somatic mutation data of LUAD patients was also downloaded from the TCGA database and used to calculate the tumor mutation burden (TMB) with the “maftools” R package. Another four independent RNA-seq data from LUAD patients, including GSE30219 (n = 85), GSE31210 (n = 226), GSE37745 (n = 106) and GSE50081 (n = 126) were downloaded from the GEO database. ERS-related gene sets (Supplementary Table 1) were downloaded from Molecular Signature Database v7.0 (MSigDB) using terms including “GO response to endoplasmic reticulum stress, GO regulation of translation in response to endoplasmic reticulum stress, GO regulation of response to endoplasmic reticulum stress”.

### Identification of lncRNAs associated with ERS

The expression correlation between the ERS-related genes and all detected lncRNAs was calculated using the Pearson test with R software based on the TCGA-LUAD and GEO GSE31210 datasets, respectively. The intersection of the lncRNAs with absolute correlation coefficient |Cor|> 0.4 and *P* < 0.001 in both the TCGA-LUAD and GSE31210 datasets were considered ERS-related lncRNAs.

### Construction of prognostic signature for LUAD

To identify ERS-related lncRNAs with prognostic significance, a univariate Cox analysis of overall survival (OS, as dependent variable) and ERS-related lncRNA expression (as independent variables) in the TCGA dataset was first performed using the “survival” R package. LncRNAs with a *P*-value < 0.05 were used for further screening of those with a prognostic signature using the Least Absolute Shrinkage and Selection Operator (LASSO) Cox regression analysis with the “glmnet” R package. The lncRNAs with non-zero coefficients were then chosen as the final ERS-related lncRNAs. They were used to construct a risk score model: (coefficient of lncRNA1 × expression of lncRNA1) + (coefficient of lncRNA2 × expression of lncRNA2) +⋯∙   + (coefficient of lncRNA_n_ × expression of lncRNA_n_).

### Validation of the prognostic model

The risk score model was used to calculate the risk score of each patient in the TCGA cohort. The TCGA cohort was then divided into HS and LS groups using the median risk score as a cutoff value. The overall risk model evaluation was accomplished by Kaplan–Meier survival analysis between two risk groups and time-dependent ROC curve analysis with the “survivalROC” R package. The area under the curve (AUC) was calculated at 1, 3, and 5 years to assess the diagnostic value of the risk signature.

To validate the ERS-related lncRNAs prognostic signature, the same method used to evaluate the TCGA dataset was applied to the three independent validation datasets (GSE30219, GSE31210, and a combined dataset from GSE37745 and GSE50081 with batch effect removing by sva R package). The LUAD patients were divided into HS and LS groups based on the median risk score calculated for each cohort by the above prognostic formula. The performance of the signature was then evaluated using Kaplan–Meier survival analysis and ROC curve analysis.

### Independent prognosis assessment of clinicopathological factors

Univariate and multivariate Cox regression analyses were used to determine which risk score and clinicopathological parameters (including age, gender, and stage) were the independent prognostic factors related to OS of LUAD patients using the TCGA dataset. The proportional hazards (PH) assumption was checked by the Schoenfeld test. Finally, results from the final multivariable Cox PHs regression were used to create a nomogram to predict the 1, 3, and 5-years OS of LUAD patients with the “rms” R package. Calibration curves were plotted to assess the accuracy of the nomogram, which shows the disparity between the predicted survival probability and the actual observed survival rates.

### Functional enrichment analysis

Differential gene expression analysis between the HS and LS groups from the TCGA cohort was conducted using the “limma” R package. A volcano plot was used to display differentially expressed genes (DEGs). DEGs were defined as genes with *P*-value < 0.05 and |FoldChange|> 2. The Kyoto Encyclopedia of Genes and Genomes (KEGG) and Gene Ontology (GO) functional enrichment of DEGs were performed by the “ClusterProfiler” R package. The KEGG pathways or GO terms with P < 0.05 were considered significantly enriched.

### Tumor mutation burden, immune infiltration and responses analysis

The TMB difference between the HS and LS groups was compared. T cell dysfunction and exclusion signature were evaluated by the TIDE model^28^. The abundance of immune cells in both HS and LS groups was estimated using CIBERSORT algorithms. The presence of stroma in tumor tissue (stromal score), the infiltration level of immune cells in tumor tissue (immune score), and tumor purity (estimate score) were predicted using the ESTIMATE algorithm based on gene expression data. Furthermore, a single sample Gene Set Enrichment Analysis (ssGSEA) analysis was conducted to quantify the immune cells and functions using the “gsva” R package. The genes' expression in immune checkpoints and human leukocyte antigen (HLA) were compared between the HS and LS groups.

### Chemotherapeutic response prediction

The Genomics of Drug Sensitivity in Cancer (GDSC) database^29^ version 2 (198 anticancer drugs against 809 cell lines) was used for the estimation of drug response. Five common drugs against non-small cell lung cancer (NSCLC) contained in GDSC were selected for chemotherapeutic sensitivity estimation, including cisplatin, docetaxel, gemcitabine, paclitaxel and vinorelbine. The IC50 of drugs was assessed using the oncoPredict package and compared between two risk groups.

### Patient recruitment and clinical sample collection

A cohort of 14 LUAD patients was prospectively recruited in 2023 at the Second Affiliated Hospital of Fujian Medical University (Quanzhou, Fujian, China). Fresh cancer tissues and adjacent non-cancerous tissues were collected from surgically resected specimens during this period. Samples were immediately frozen in liquid nitrogen following resection and subsequently stored at − 80 °C until RNA extraction.

### Cell cultures

Two LUAD cell lines, H1299 and A549, were obtained from the cell repository of the American Type Culture Collection (ATCC) and used for cell experiments. Cells were cultured in Dulbecco’s modified Eagle’s medium (DMEM) supplemented with 10% fetal bovine serum (FBS) and 1% penicillin/streptomycin sulfate at 5% CO_2_ and 37°C.

### Cell transfection

LncRNA RP11-295G20.2 lentivirus was constructed from the Gikai gene (Shanghai, China). RP11-295G20.2-targeting short hairpin RNAs (shRNAs, RP11-295G20.2-shRNA1 and RP11-295G20.2-shRNA2) were synthesized, and transfected into both H1299 and A549 cells by Lipofectamine 2000. The targeted sequences of RP11-295G20.2 shRNAs are: RP11-295G20.2-shRNA1: 5′-AAGCCTCACAGCATGCACTGTTACT-3′; and RP11-295G20.2-shRNA2: 5′-GACCTGAGATCTGTGTGATCGT-3′. Detailly, cells were plated for 24 h. The plasmid and transfection reagent were added with FBS-free DMEM and incubated for 10–15 min at room temperature. Then, the transfection complex was added to cell cultures and replaced with DMEM supplemented with FBS after 10–12 h. Cells were collected after 48 h, and their transfection efficiency was determined by RT‒qPCR.

### Cell proliferation, migration and invasion assays

Cell Counting Kit-8 (CCK-8), wounding healing and transwell invasion assays detailly described as follows were conducted to measure cell proliferation, migration and invasion abilities.

CCK-8 assay: 2 × 10^4^ cells were seeded in a 96-well plate in triple replication for each group and cultivated for 24, 48, and 72 h. Ten μL CCK-8 solution was added to each well and incubated for 2 h at 37°C followed by absorbance measuring. The absorbance was measured at 450 nm with a microplate reader.

Wounding healing assay: cells were cultivated at 90% confluence in a well. The dislodged cellular debris in the well was washed using PBS. Then, a 10 µL pipette tip was used to make scratches. Cells were incubated in FBS-free DMED for 48 h, and photographed with a microscope.

Transwell invasion assay: cells (2 × 10^4^ cells/well) were placed in an invasion chamber with FBS-free DMED. DMED supplemented with 10% FBS was added to the lower chamber. The Transwell cells were cultured at 37°C and 5% CO2 for 48 h. The invasive cells were fixed with methanol and stained with crystal violet (10 min). Cells on the upper chamber surface were wiped off using cotton swabs. The invasive cell number in the lower chamber was calculated at six spots randomly photographed under a microscope.

### RNA extraction and RT‒qPCR

Total RNA including mRNA and lncRNA of cells was extracted using Tissue RNA Purification Kit (RN002plus, ES Science, China). RNA was reverse-transcribed into cDNA using the PrimeScript™ RT Reagent kit (Tli RNaseH Plus, Takara, China). The gene expression level of lncRNA RP11-295G20.2 and an ERS biomarker GPR78^9,30,31^ were measured using real-time qPCR with the SYBR Premix Ex Taq™ Kit (Takara, Dalian, China) on Gentier 96 Real-Time PCR System (Tian Long, China). The primer sequences of RP11-295G20.2 were: forward 5′-GTACTATTGCGGCCGCCCTC-3′; reverse 5′-AATCAAACTTGAAAGTGGGA-3′. The primer sequences of GRP78 were: forward 5′-CTGGAACTATTGCTGGCCTA-3′; reverse 5′-TGGTGAGAAGAGACACATCG-3′. The GAPDH was used as the internal reference, with primers: forward 5′-GCGGGGCTCTCCAGAACATCAT-3′ and reverse 5′-CCAGCCCCAGCGTCAAAGGTG-3′. The relative expression of RP11-295G20.2 and GPR78 were calculated using the 2^−ΔΔCt^ method.

### Statistical analysis and graphing

For pairwise comparisons, the Wilcoxon signed rank-sum test was used. Two-way ANOVA was used to analyze the difference of the CCK-8 assay. Statistical analysis and plotting were performed using R software. The heatmap of gene expression was visualized using the R package “pheatmap”. Forest plots displayed the prognostic lncRNAs associated with OS using the R package “forestplot”. PCA analysis was performed using the ‘prcomp’ R package. The Sankey diagram, which depicted the relationship between the mRNAs and ERS-related lncRNAs as well as two risk groups, was plotted using the ggalluvial software.

### Ethics approval and consent to participate

Clinical sample acquisition and subsequent analyses were approved by the Research Ethics Committee of the Second Affiliated Hospital of Fujian Medical University and donors’ Consent. All volunteers participating in this study gave their informed consent. Procedures followed in this study were under the ethical standards of concerned institutional policies (No. 515/Year 2023).

## Results

### Characterization of ERS-related lncRNAs in LUAD

**Figure 1:**
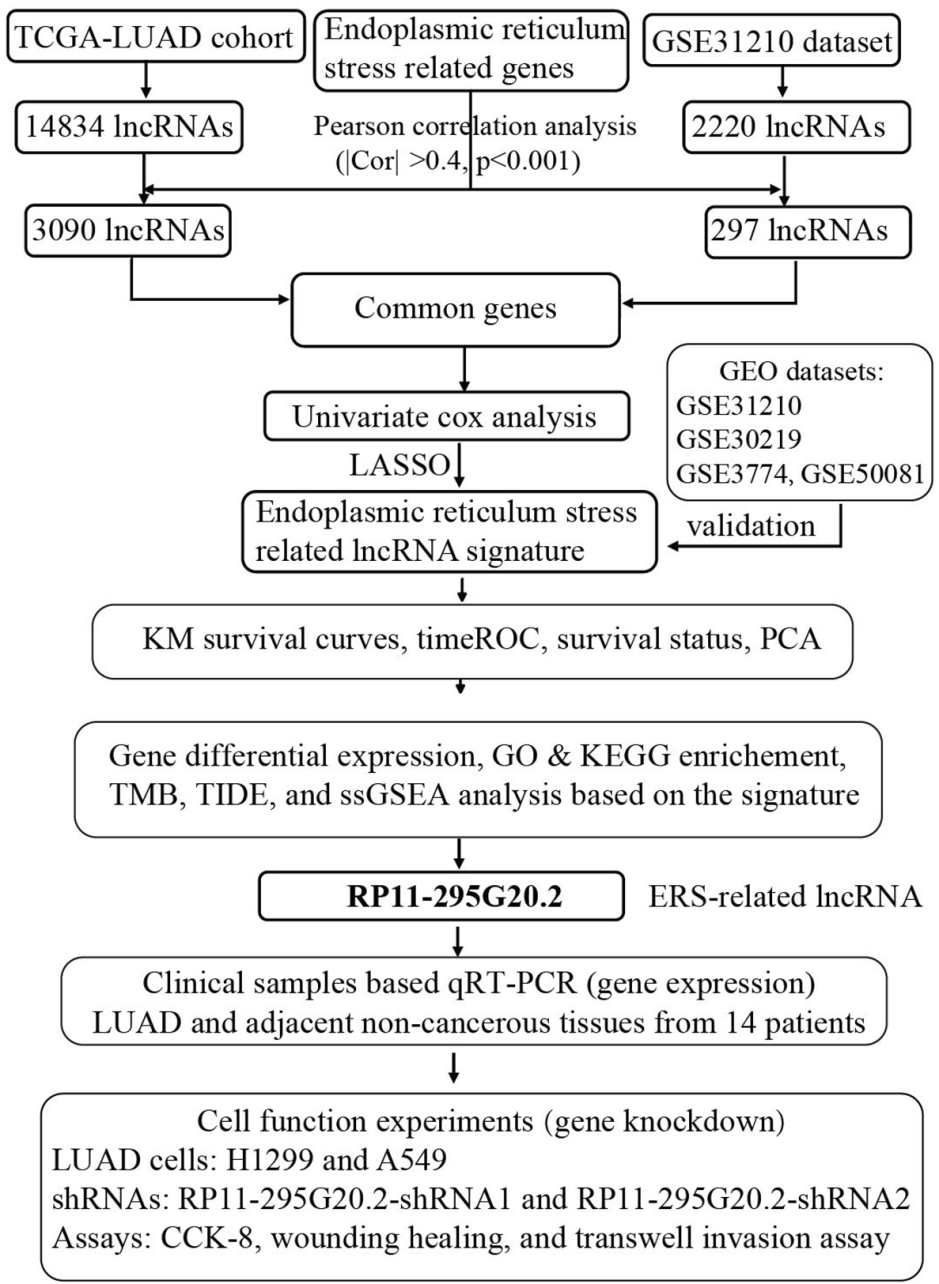
Workflow diagram of this study.

The analysis flow chart of our study was shown in Fig. 1. We detected 14834 expressed lncRNAs in the TCGA dataset and 2220 in the GSE31210 dataset. Among them, 3090 and 297 lncRNAs were highly correlated to the ERS-related genes in the TCGA and GSE31210 datasets, respectively, contributing to 155 shared ERS-related lncRNAs.

### Nine ERS-related lncRNAs based signature for LUAD

**Figure 2:**
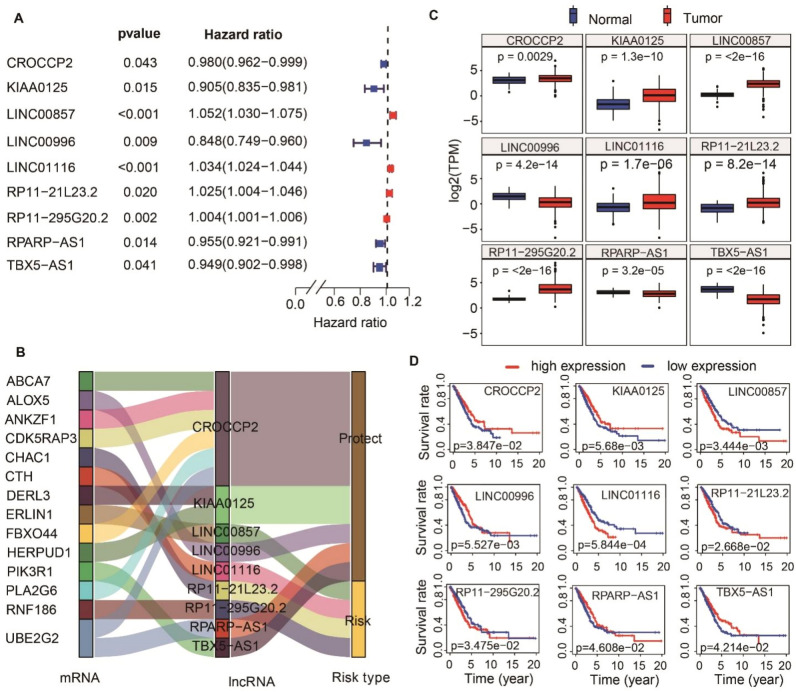
Construction of nine ERS-related lncRNAs signature. (**A**) Forest plots showing the results of the univariate Cox regression analysis between nine lncRNAs expression and OS. (**B**) The Sankey diagram displays the relationship between the mRNAs and ERS-related lncRNAs classified as the risk or protective subtypes. (**C**) Comparisons of the expression profiles of ERS-related lncRNAs in TCGA. The blue represents the gene expression in normal tissues, while the red represents the gene expression in tumor tissues. (**D**) Survival analysis of the nine ERS-related lncRNAs in TCGA databases.

Univariate Cox analysis revealed 13 significant lncRNAs (Fig. S1). Six lncRNAs showed higher expression in LUAD tissues than in normal tissues, whereas seven exhibited lower expression in tumor tissues (Fig. S1). The LASSO Cox regression analysis based on the 13 significant lncRNAs yielded nine prognostic lncRNAs, among which five (CROCCP2, KIAA0125, LINC00996, RPARP-AS1, and TBX5-AS1) were protective factors and four (LINC00857, LINC01116, RP11-21L23.2, and RP11-295G20.2) were risk factors (Fig. S1; Fig. 2A,B). The nine lncRNAs targeted 14 ERS-related genes and displayed significant differences in gene expression between normal and tumor tissues (Fig. 2C). Furthermore, survival analysis results revealed significant differences in OS between high and low expression groups divided by each of the nine ERS-related lncRNAs (Fig. 2D). The risk score model based on the nine ERS-related lncRNAs was constructed as follows: (−0.003 × expression of CROCCP2) + (−0.032 × expression of KIAA0125) + (0.033 × expression of LINC00857) + (−0.021 × expression of LINC00996) + (0.026 × expression of LINC01116) + (0.014 × expression of RP11-21L23.2) + (0.002 × expression of RP11-295G20.2) + (−0.020 × expression of RPARP-AS1) + (−0.004 × expression of TBX5-AS1).

**Figure 3:**
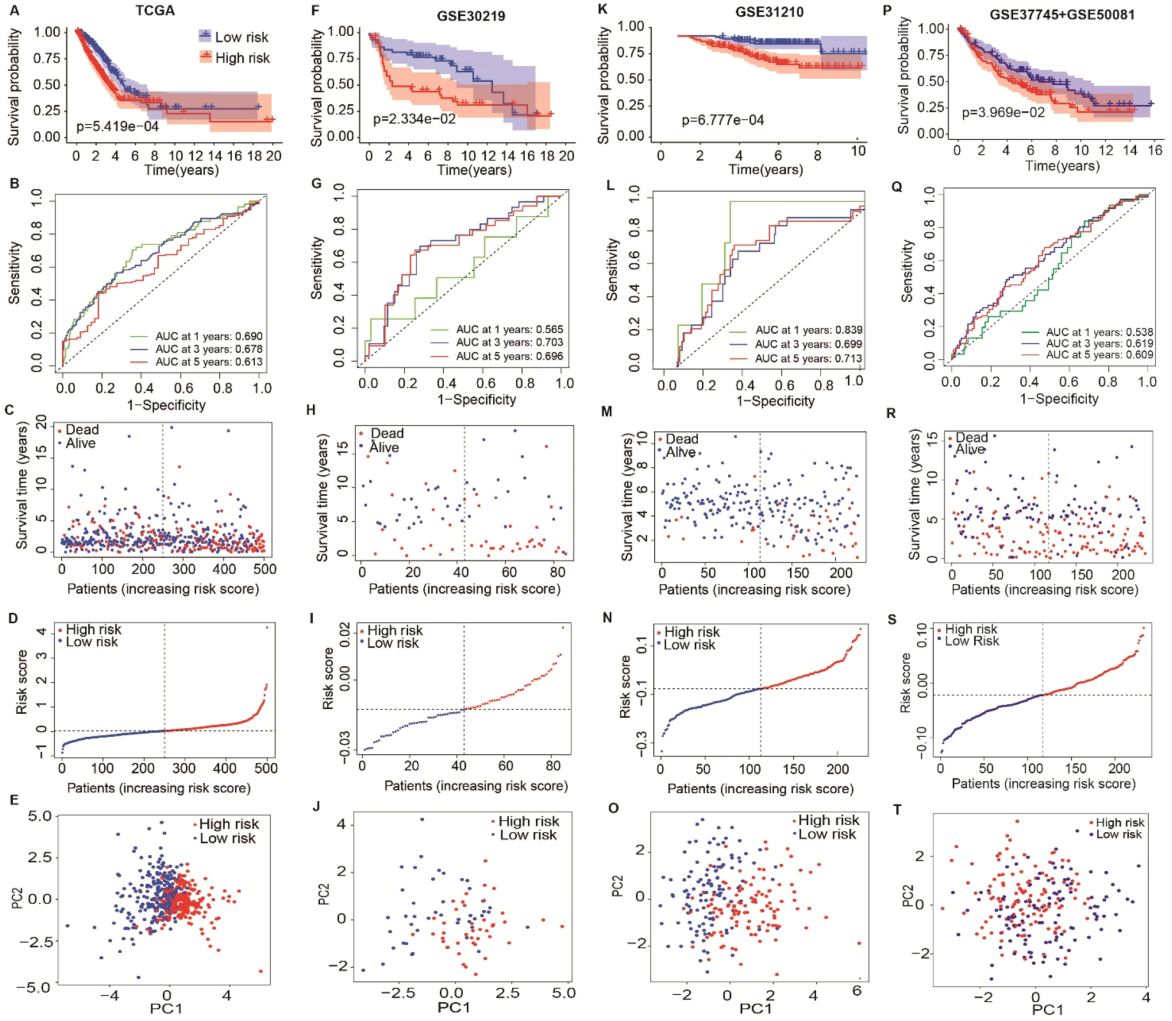
The prognostic value of the risk signature was constructed by nine endoplasmic reticulum stress (ERS) related lncRNA in the training and validation sets. Kaplan Meier survival analysis (**A**), AUC of ROC at 1-, 3- and 5-year OS (**B**), distributions of OS status, OS and risk score (**C**), distribution and median value of the risk scores (**D**) and principal component analysis (PCA) (**E**) in the TCGA cohort. Kaplan Meier survival analysis (**F**, **K**, **P**), AUC of ROC at 1-, 3- and 5-year OS (**G**, **L**, **Q**), distributions of OS status, OS and risk score (**H**, **M**, **R**), distribution and median value of the risk scores (**I**, **N**, **S**) and PCA (**J**, **O**, **T**) between HS and LS groups in the training sets including GSE30219, GSE31210, and combined GSE37745 and GSE50081 datasets.

LUAD patients were divided into HS and LS groups based on the cutoff of the median risk score in the TCGA (Fig. 3A–E) and several GEO cohorts ((Fig. 3F–T). The survival times were longer in the LS group than the HS group in the TCGA (*P* = 5.419e-04), GSE30219 (*P* = 2.334e-02), GSE31210 (*P* = 6.777e-04), and combined datasets of GSE37745 + GSE50081 (*P* = 3.969e-02) cohorts (Fig. 3A,F,K,P). The areas under the ROC curve (AUC) of the TCGA dataset were 0.690, 0.678 and 0.613 at 1, 3, and 5 years of survival, respectively (Fig. 3B). For the cohorts of validation datasets, the AUC were 0.565, 0.703, and 0.696 for 1, 3, and 5 years survival in the GSE30219, 0.839, 0.699 and 0.713 for 1, 3, and 5 years survival in GSE31210, and 0.538, 0.619 and 0.609 for 1, 3, and 5 years survival in the GSE37745 + GSE50081 dataset (Fig. 3G,L,Q). In these four cohorts, the HS group contained much more patients who were already dead or had a shorter survival time. In contrast, the LS group had more patients who were still alive or had a longer survival time (Fig. 3C,H,M,R).

### Independent prognostic value of the ERS-related lncRNA signature

**Figure 4:**
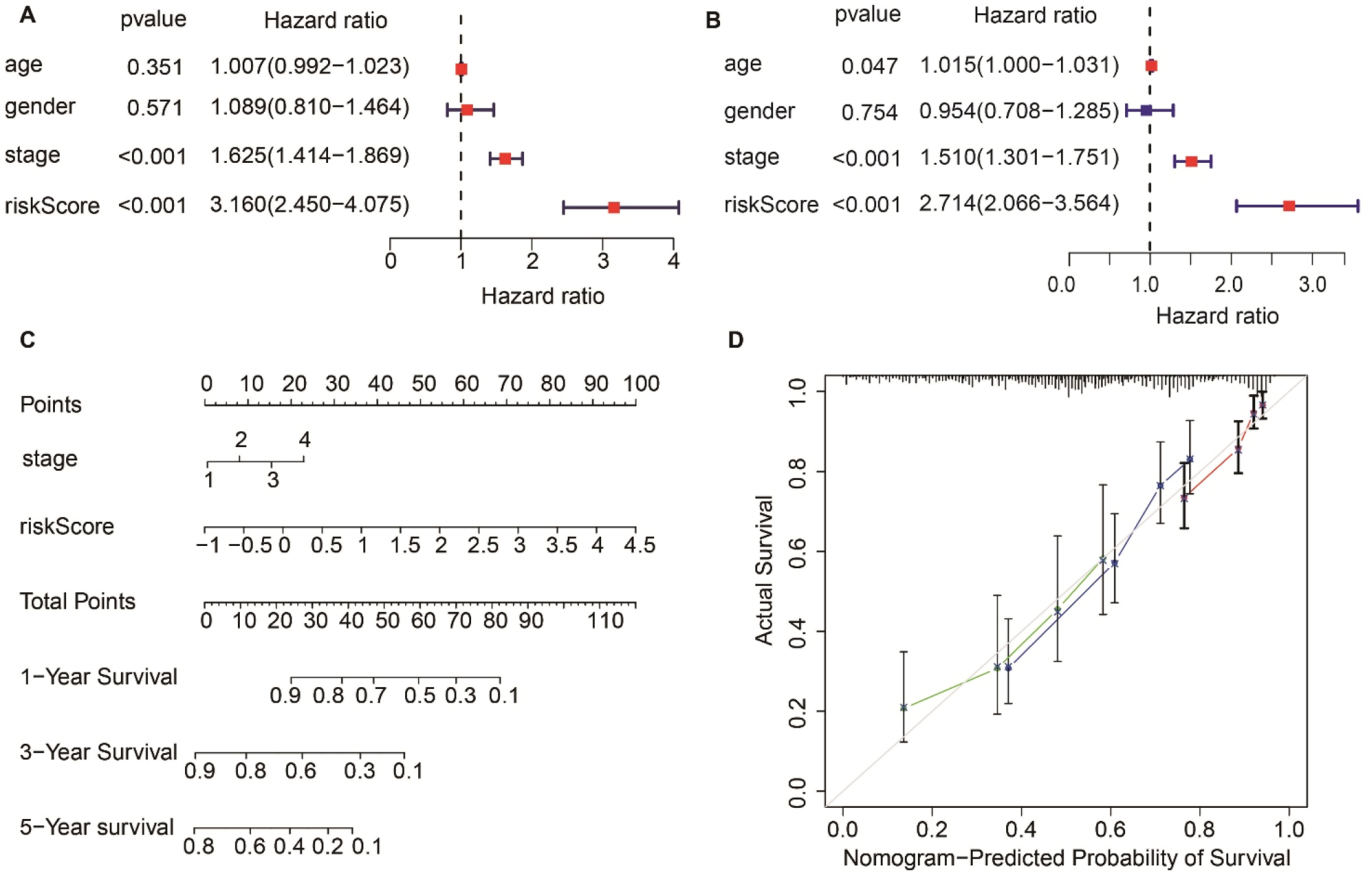
Estimation of the prognostic accuracy of the ERS-related lncRNA prognostic signature and other clinicopathological variables in the LUAD patients. (**A**) Univariate Cox regression analysis shows the correlation between overall survival and clinicopathological parameters such as age, gender, stage and the ERS-related lncRNA prognostic signature risk score. (**B**) Multivariate Cox regression analysis reveals that stage and risk score (*P* < 0.001) are independent prognostic indicators for the overall survival rates of LUAD patients. (**C**) The prognostic nomogram constructed using the risk score from ERS-related lncRNA prognostic signature and clinicopathological stage predicts 1-, 3-, 5-year survival rates of LUAD patients. (**D**) Calibration curves display the concordance between predicted and observed 1-year (red line), 3-year (blue line), and 5-year (green) survival rates of LUAD patients based on the prognostic nomogram after bias correction.

The univariate and multivariate Cox regression analysis revealed that the stage and risk score were independent prognostic indicators (*P* < 0.001, HR > 1) of OS for LUAD patients in the TCGA dataset (Fig. 4A,B). The PH assumption was met by the model as the Schoenfeld test demonstrated (p = 0.63) (Fig. S2). Simultaneously, a prognostic nomogram based on the two independent prognostic factors was constructed to predict 1-, 3-, and 5-year survival rates of LUAD patients (Fig. 4C). Calibration curves demonstrated consistency between predicted and observed survival rates, indicating the good performance of the prognostic nomogram (Fig. 4D).

### Correlation of the prognostic signature with clinicopathological features, somatic mutation and TIDE

To unravel the relationship between the risk score of ERS-related lncRNAs and other clinicopathological factors, we compared the differences between groups classified by gender, stage, and age. Results showed that the male had a significantly higher risk score than the female (*P* < 0.001, Fig. S3A). Age does not affect the risk score (Fig. S3B). The risk score increased as the disease stages progressed evidenced by higher risk scores in stages III and IV than in stages I&II (Fig. S3C). Moreover, we discovered that the HS group had higher TIDE and TMB than the LS group (Fig. S3D,E). And there was a positive correlation between TMB and risk score (Fig. S3F).

### Functional enrichment of differentially expressed genes between HS and LS groups

**Figure 5:**
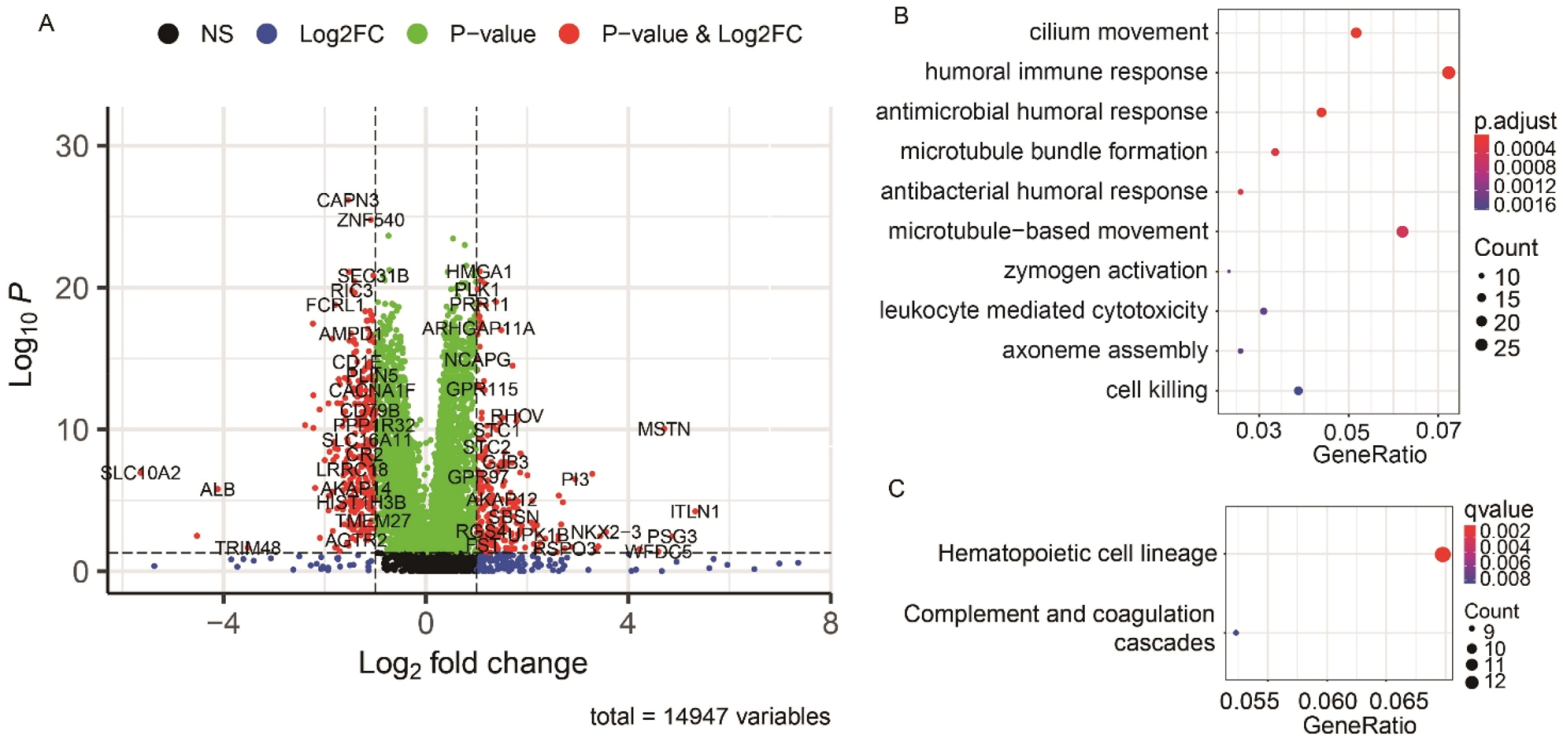
Functional annotation and pathway enrichment analysis between HS and LS groups of the TCGA cohort. (**A**) Volcano plot of DEGs between HS and LS groups. (**B**) The GO enrichment of DEGs. (**C**) The KEGG enrichment of DEGs.

Differential gene expression analysis revealed 482 DEGs between HS and LS groups, including 191 up-regulated and 291 down-regulated mRNAs in the HS groups (Fig. 5A). These DEGs were functionally enriched in 10 biological processes GO terms (*P* < 0.001, Fig. 5B): cilium movement, humoral immune response, antimicrobial humoral response, microtubule bundle information, antibacterial humoral response, microtubule-based movement, zymogen activation, leukocyte mediated cytotoxicity, axoneme assembly and cell killing. KEGG enrichment results showed that the DEGs were functionally enriched in “hematopoietic cell lineage” and “complement and coagulation cascades” pathways (Fig. 5C).

### Distinct immune infiltration levels, immune responses and chemosensitivity between HS and LS groups

**Figure 6:**
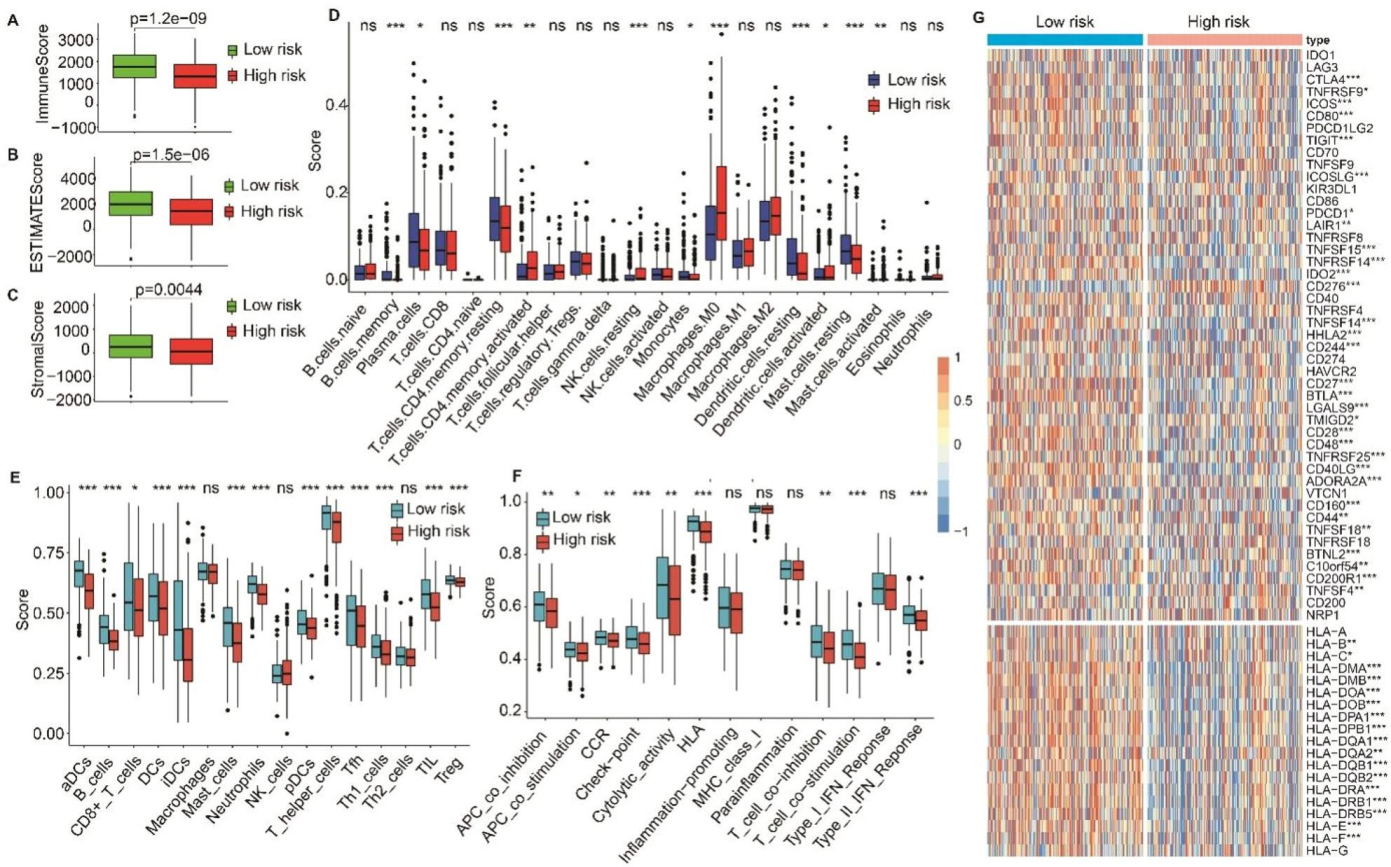
Immune infiltration analysis in the HS and LS groups. (**A**–**C**) The prognostic significance of immune score, stromal score, and estimate score by ESTIMATE algorithm. (**D**, **E**) Boxplot for ssGSEA scores [immune cells scores (**D**) and immune functions scores (**E**)]. (**F**) Immune cell content evaluated by CIBERSORT. (**G**) Heatmap of gene expression for immune checkpoints and human leukocyte antigen (HLA) genes. Adjusted *P* values were showen as: ns, not significant; *P < 0.05; **P < 0.01; ***P < 0.001.

**Figure 7:**
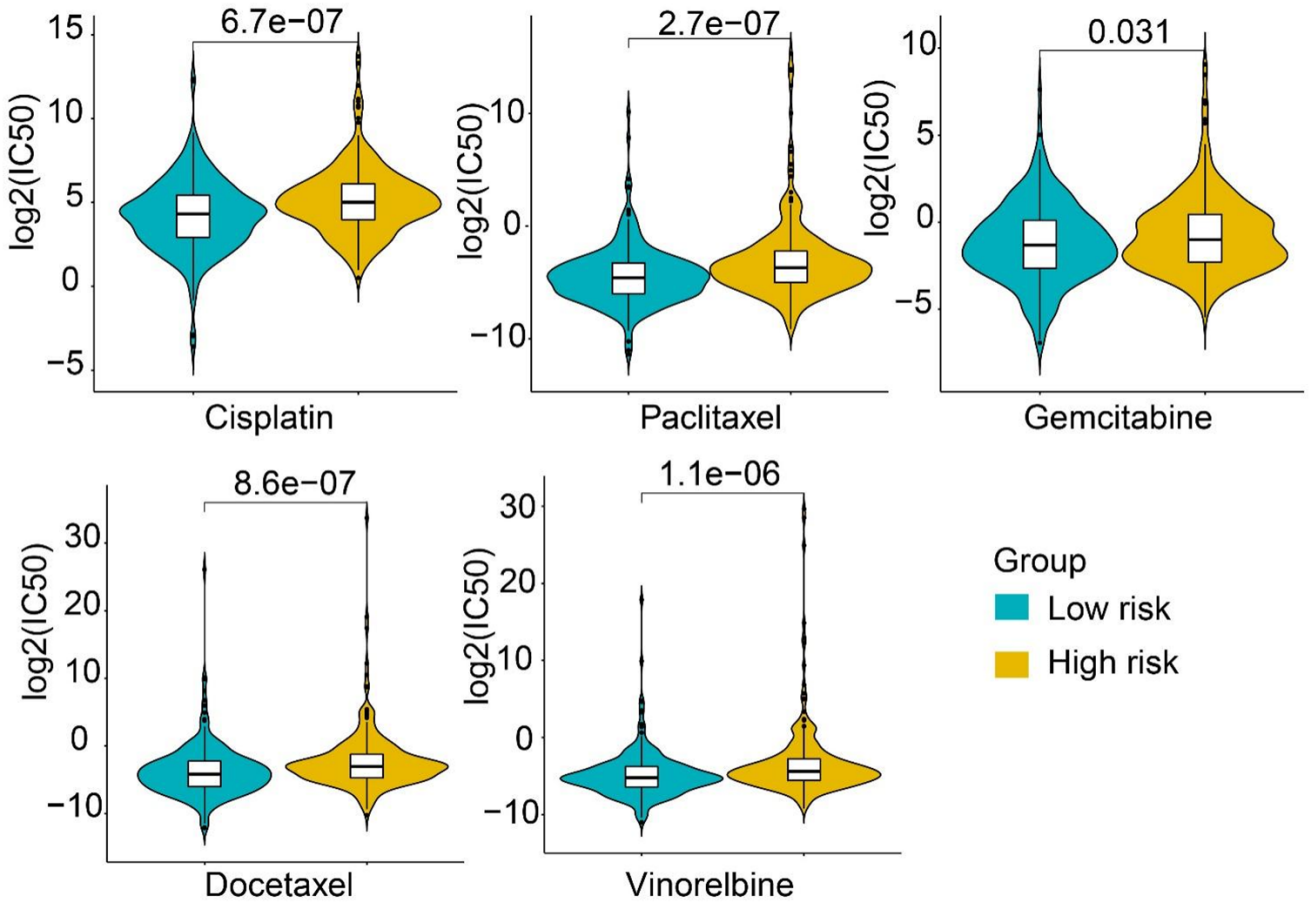
Comparisons of drug sensitivity between HS and LS groups. Box plots show the log-transformed IC50 values for five chemotherapeutic drugs between the two groups, including cisplatin, docetaxel, gemcitabine, paclitaxel and vinorelbine.

Three ESTIMATE immune scores were higher in the LS group than in the HS group (*P* < 0.05, Fig. 6A–C), demonstrating that the LS group had higher immunogenicity and immune abilities in the tumor microenvironment. The immune cell scores evaluated by CIBERSORT revealed that 11 cell types differed significantly between the two risk groups. B cells memory, plasma cells, T cells CD4 memory resting, monocytes, dendritic cells resting and mast cell resting scored higher in the LS group. In contrast, T cells CD4 memory activated, NK cells resting, macrophage M0, dendritic cell activated and mast cell activated scored higher in the HS group (Fig. 6D). ssGSEA analysis exhibited that scores of 13 cell types were significantly different in the two groups, including B cells, Treg and T helper cells (Fig. 6E). Specifically, nine immune functions displayed significantly higher scores in the LS group than in the HS group (Fig. 6F), including immune checkpoints and HLA. To further evaluate the immune response in the two groups, we identified genes involved in immune checkpoints and HLA. We found that most of the immune checkpoint genes (32/47) and HLA genes (17/19) showed higher gene expression in the LS group than in the HS group (Fig. 6G). Moreover, chemosensitivity analysis showed that significantly lower IC50 values of cisplatin, docetaxel, gemcitabine, paclitaxel and vinorelbine were in the LS group compared to the HS group (Fig. 7), suggesting that patients in the LS group were more sensitive to the five targeted drugs.

### RP11-295g20.2 is up-regulated in LUAD tissues and its knockdown suppresses LUAD cell ERS, proliferation, invasion and migration

**Figure 8:**
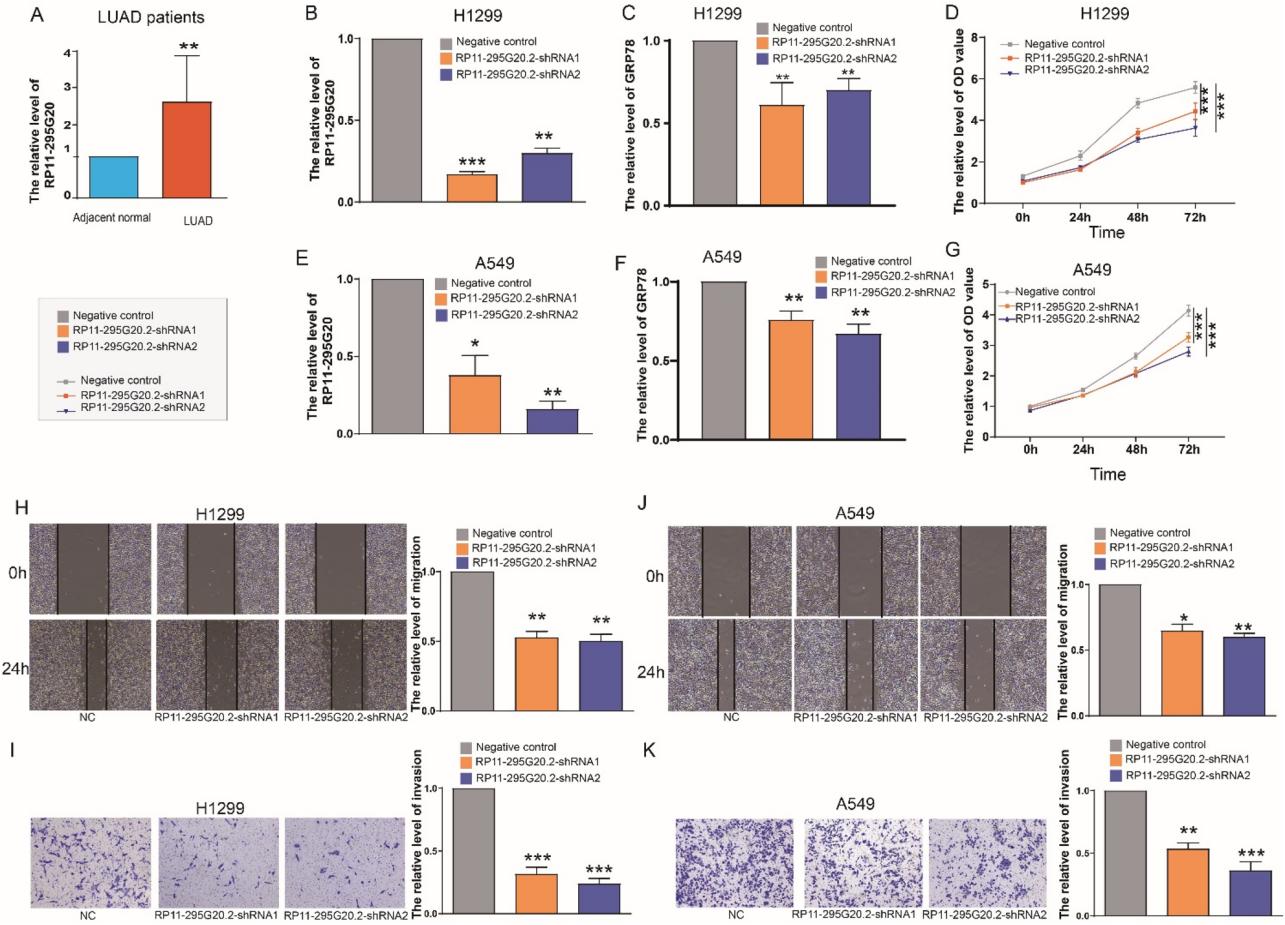
Clinical expression of RP11-295G20.2 and effects of its knockdown on LUAD cell ERS, proliferation, invasion and migration. (**A**) Gene expression differences of RP11-295G20.2 between LUAD tissues and adjacent non-cancerous tissues from LUAD patients (n = 14). (**B**) The RP11-295G20.2 and (**C**) GRP78 expression quantified by RT-qPCR in the H1299 cell line after transfecting RP11-295G20.2-shRNAs. (**C**) and (**D**) The proliferation capacity of H1299 measured by CCK-8 assay. (**E**) The RP11-295G20.2 (**F**) and GRP78 expression quantified by qRT-PCR in the A549 cell line after transfecting RP11-295G20.2-shRNAs. (**G**) The proliferation capacity of A549 measured by CCK-8 assay. (**H**) The migration capacity of H1299 cells measured by wound healing assay. (**I**) The invasive capacities of H1299 cells evaluated by transwell assays. (**J**) The migration capacity of A549 cells measured by wound healing assay. (**K**) The invasive capacities of A549 cells evaluated by transwell assays. *P < 0.05; **P < 0.01; ***P < 0.001.

To gain insights into mechanisms of ERS-related lncRNAs in LUAD, we selected RP11-295G20.2 as a representative candidate for in-depth functional characterization in modulating LUAD progression. RP11-295G20.2 was identified as a risk factor (hazard ratio > 1) and exhibited the most significant upregulation in LUAD tissues compared to normal samples (Fig. 2A,B).. By collecting clinical samples from LUAD patients, we performed RT-qPCR and confirmed that RP11-295G20.2 mRNA expression was significantly upregulated in LUAD tissues compared to matched normal lung tissues (Fig. 8A).

To elucidate the functional role of RP11-295G20.2 on LUAD ERS and progression, we conducted loss-of-function experiments utilizing small interfering RNA (siRNA) to knock down RP11-295G20.2 expression in LUAD cell lines (Fig. 8B–K). RT-PCR showed that RP11-295G20.2 expression was significantly lower in H1299 and A549 cells transfected with two shRNAs (RP11-295G20.2-shRNA1 and RP11-295G20.2-shRNA2) than normal cells (Fig. 8B,E), suggesting successful transfection of the shRNAs into LUAD cells. GRP78 is an ERS biomarker that is induced under ERS conditions and acts as an anti-apoptotic role in cancer cells^9,30,31^. To better explore relationship between RP11-295G20.2 and ERS response, we assessed changes in the ERS-related gene GRP78 in RP11-295G20.2 knockdown cells. RT-qPCR results showed that GRP78 expression was significantly down-regulated in both RP11-295G20.2-shRNA1 and RP11-295G20.2-shRNA2 cells compared to the negative control cells (Fig. 8C,F). CCK-8 assay indicated that RP11-295G20.2 knockdown significantly suppressed LUAD cell growth, as evidenced by markedly reduced absorbance values in RP11-295G20.2-shRNA1 and RP11-295G20.2-shRNA2 cells (Fig. 8D,G). Wounding healing and transwell invasion assays then demonstrated that RP11-295G20.2 knockdown strongly inhibited cell immigration and invasion capabilities in vitro (Fig. 8H–K). These results suggested that RP11-295G20.2 knockdown influenced ERS and suppressed cell progression of LUAD cells.

## Discussion

LUAD is known for a high degree of heterogeneity, genetic mutation and epigenetic modification, which increases its treatment complexity and effectiveness. Molecular biomarkers and new targets for individualized diagnosis and prognosis of LUAD are still lacking. With the development of precise medicine and bioinformatics, signatures for predicting the clinical outcome and aiding immunotherapy in LUAD were urgently needed and developed, including lncRNA signatures for LUAD derived by genome instability^32^, immune^33,34^, pyroptosis^35^, redox^36^ and so on. ERS and the unfolded protein response (UPR) are highly induced in tumors and closely associated with cancer cell survival due to various stress conditions in the tumor microenvironment. In recent years, research efforts have focused on developing approaches to exploit ERS mechanisms for cancer therapy. ERS was aggravated in cancer cells by targeting UPR-related genes to enhance apoptosis and trigger tumor cell death. Additionally, lncRNAs involved in tumorigenesis and metastasis are being studied for their roles in cancer diagnosis and therapy^37^. Misexpression of lncRNAs confers the cancer cell capacities for initiation, growth, and metastasis^37^. Many lncRNAs have been implicated in regulating ERS in cancer cells and are assumed to be tumor biomarkers and therapeutic targets^22,38^. Revealing the ERS-related lncRNAs in LUAD will provide new insights into understanding tumorigenesis and therapeutic strategies.

In this study, we developed a nine ERS-related lncRNAs based signature with good prognostic performance in LUAD patients from the TCGA and GEO cohorts, which serves as a new biomarker for LUAD prognostic prediction and treatment. We also construct a nomogram based on the ERS-related lncRNAs and tumor stage with significant independent prognosis to provide a model for predicting the OS of LUAD patients. The results showed that the nine ERS-related lncRNAs demonstrated independent prognostic ability. The most significant protective factor, LINC0996, was an immune-related lncRNA proposed as a potential target of tumor immunology in adenocarcinoma^39^. The second significantly protective KIAA0125 expression was significantly higher in LUAD tissues than in normal tissues, confirming the findings of Liu et al.^40^. Functional studies revealed that KIAA0125 suppression strongly inhibits gallbladder cancer cell migration and invasion^41^, whereas its overexpression suppressed colorectal cancer cell proliferation, migration and invasion^42^. TBX5-AS1 was closely associated with tumorigenesis and cancer progression, such as LUAD^43^. This lncRNA was down-regulated in NSCLC patients, and its overexpression inhibited cell viability, colony formation, migration and invasion^44^. RPARP-AS1 was an N-6 methylation-related lncRNA associated with LUAD prognosis^45^. The mutation frequency of the protective CROCCP2 was high^46^, but its role in LUAD is unknown. For the four risk lncRNA factors, elevated LINC0857 expression promoted pancreatic cancer cell proliferation and mobility by decreasing miR-130b expression^47^. In NSCLS, higher RP11-21L23.2 expression was associated with a shorter OS^24,48^. The up-regulated lncRNA RP11-295G20.2 promoted cell growth and inhibited autophagy in hepatocellular carcinoma cells by targeting the tumor suppressor PTEN to lysosomal degradation^21^. Our cell proliferation, migration and invasion assays supported that the suppression of RP11-295G20.2 inhibited cell abilities of proliferation, invasion and migration. GRP78, also known as HSPA5, is an ER molecular chaperone and a major target induced by the unfolded protein response (UPR)^30^. Overexpression of GRP78 enhances ERS capacity and protects cancer cells from apoptosis^30,31^. As GRP78 is induced under ERS conditions and anti-apoptotic in cancer cells, we hypothesized that knockdown of risk factor RP11-295G20.2 would down-regulate GRP78 expression if it modulates ER stress. Our results showed that RP11-295G20.2 inhibition indeed led to down-regulation of GRP78, providing evidence that RP11-295G20.2 knockdown alleviated ERS in LUAD cells. However, the molecular mechanism of RP11-295G20.2 in promoting LUAD needs further investigation in the future, for example, using RNA sequencing technology and additional biological assays. The remaining risk factor LINC116 in LUAD is aslo unexplored and deserves to be investigated in the future.

Because of the complexities of LUAD heterogeneity, biomarkers and targets are required for precision therapeutics accomplished by unrevealing the molecular mechanisms of the regulation of LUAD. We discovered the potential roles of ERS-related lncRNAs in LUAD by comparing the differences in gene function, cell immunity and mutation between the HS and LS groups. DEGs between HS and LS groups were found to be enriched in cell movement-related pathways such as cilium movement, microtubule bundle information and microtubule-based movement; immunity pathways such as the humoral immune response, hematopoietic cell lineage, and complement and coagulation cascades; cell deaths related pathways including leukocyte mediated cytotoxicity and cell killing. It is well known that ERS can dynamically reprogram function of immune cells^49^ and trigger different model of cell death^50^. Interestingly, the functional enrichment analysis in our study revealed that the most enriched pathways were related to microtubules and cilia. ER dynamics can drive the formation of microtubule bundles that play a crucial role in cell cycle and mobility^51^. Cilia are microtubule-based organelles that critically control proliferation by mediating cell-extrinsic signals and by regulating cell cycle entry^52^. The aberrant expression of microtubule proteins and abnormal functions of cilia have been demonstrated to contribute to oncogenesis and cancer progression^52,53^, including lung cancer^53–55^. These findings suggested that ERS-related lncRNAs may predominantly regulate LUAD progression through influencing LUAD cell processes associated with microtubule and cilium functions, including cell migration and cell cycle regulation, as well as LUAD progression by modulating cell death and immune responses. Immune checkpoint inhibitors (ICIs), which activate T cells to kill tumors, are a type of immunotherapy widely used to treat various types of cancer^56^. The numerous higher expressed HLA genes indicated the more active anti-tumor immune microenvironment in the LS group. A previous study reported that the LUAD subgroup with higher immune checkpoints gene scores consisted of patients with increased expression of immune checkpoints genes, favorable survival outcomes and enhanced immune cell infiltration^38^. Consistently, the evaluated gene expression of immune checkpoints in the LS group favored their increased immune cell infiltration and favorable survival outcomes. TMB is a positive biomarker for predicting clinical response to ICIs and screening cancer patients suitable for ICIs treatment^34^. Cancer patients with a high TMB have a better survival chance if they receive immunotherapy^57^. Despite higher TIDE and later stages of HS patients, which indicates poor immune ability, the high TMB suggests that those HS patients are suitable for immunotherapy. Infiltrating immune cells in the tumor microenvironment largely determined cancer progression and immunotherapy response. HS LUAD patients had higher immunosuppression suggested by higher NK cells infiltration and lower immunoreactivity, evidenced by lower fractions of B cells, CD8^+^ cells, macrophages, neutrophils, mast cells, and helper T cells than LS patients, resulting in a poorer prognosis and tumor progression. The different IC50 values of five commonly used drugs (cisplatin, docetaxel, gemcitabine, paclitaxel and vinorelbine) in the HS and LS groups supported that patients in different risk groups confer distinct resistance to targeted cancer therapies.

Our model has been validated to perform well on three independent datasets and can serve as a biomarker for LUAD patients. The potential limitations of our study should be acknowledged. More robust validation and clinical application of the model will benefit from further validation of the signature in large-scale cohorts. Functional experiments will help confirm the relationship between the ERS-related lncRNAs and tumor immune microenvironment and cell deaths in LUAD.

## Conclusion

We constructed an accurate prognostic signature consisting of nine ERS-related lncRNA. The signature serves as a new biomarker to assess distinct OS, immune cell level in the tumor microenvironment, TMB, the potential immunotherapeutic benefits, and chemotherapeutic sensitivity of commonly used drugs (cisplatin, docetaxel, gemcitabine, paclitaxel and vinorelbine) for LUAD patients. The knockdown of lncRNA RP11-295G20.2 changed ERS and suppressed cell proliferation, invasion and migration in LUAD cells. Our findings shed light on the roles of ERS-related lncRNAs in clinical implications and progression of LUAD.

### Supplementary Information

Supplementary Table S1.

Supplementary Figures.

## Data Availability

Datasets used in this study were collected from publicly available databases including the Cancer Genome Atlas database (https://portal.gdc.cancer.gov/) and the Gene Expression Omnibus database (https://www.ncbi.nlm.nih.gov/geo/).
