# Supplementary material for: Characterization of an endoplasmic reticulum stress-associated lncRNA prognostic signature and the tumor-suppressive role of RP11-295G20.2 knockdown in lung adenocarcinoma
Source: Sci Rep. 2024 May 29;14:12283. doi: 10.1038/s41598-024-62836-z (PMC11137026; doi:10.1038/s41598-024-62836-z)
Supplement: Supplementary file 2 — Supplementary Figures. [file 41598_2024_62836_MOESM2_ESM.docx]

**Supplementary Figures**

**
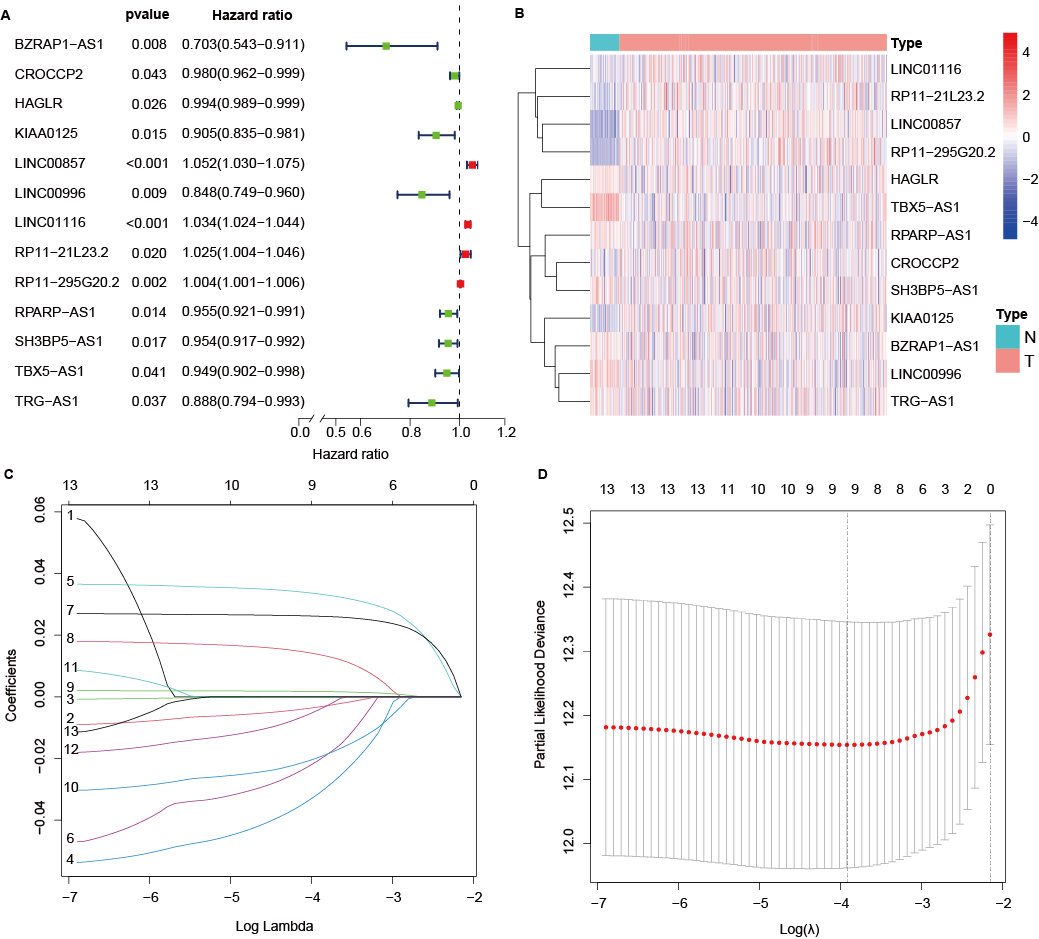
**

**Supplementary Figure 1. Construction and validation of the ERS-related lncRNA signature.**(A) The univariate analysis of ERS-related lncRNA for LUAD patients in TCGA. (B) The heatmap of ERS-related lncRNAs. (C)The selection of risk factors by LASSO analysis.


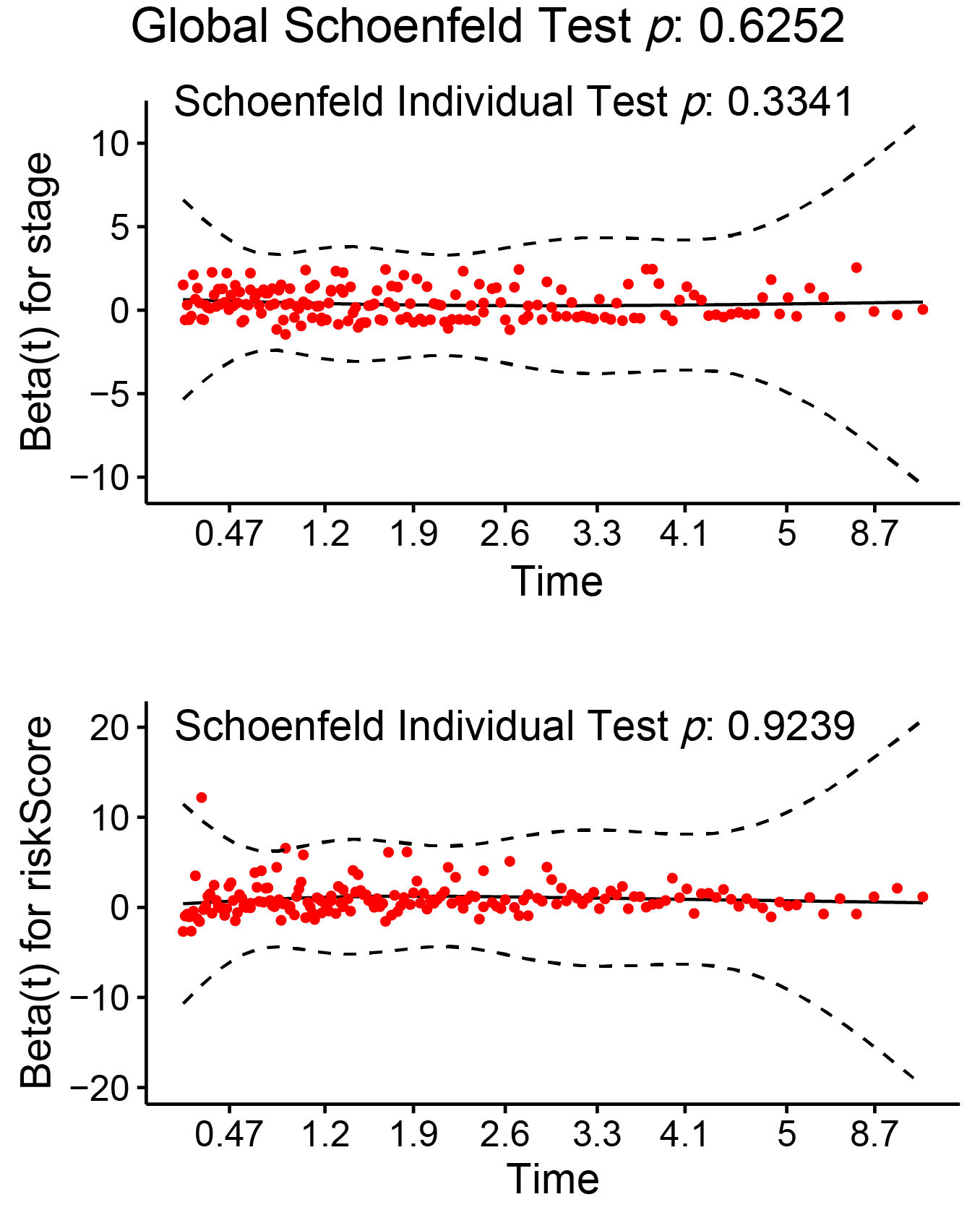


**Supplementary Figure 2.** The PH assumption demonstrated by the Schoenfeld test identifies significant prognostic factors for the construction of the survival-prediction nomogram.


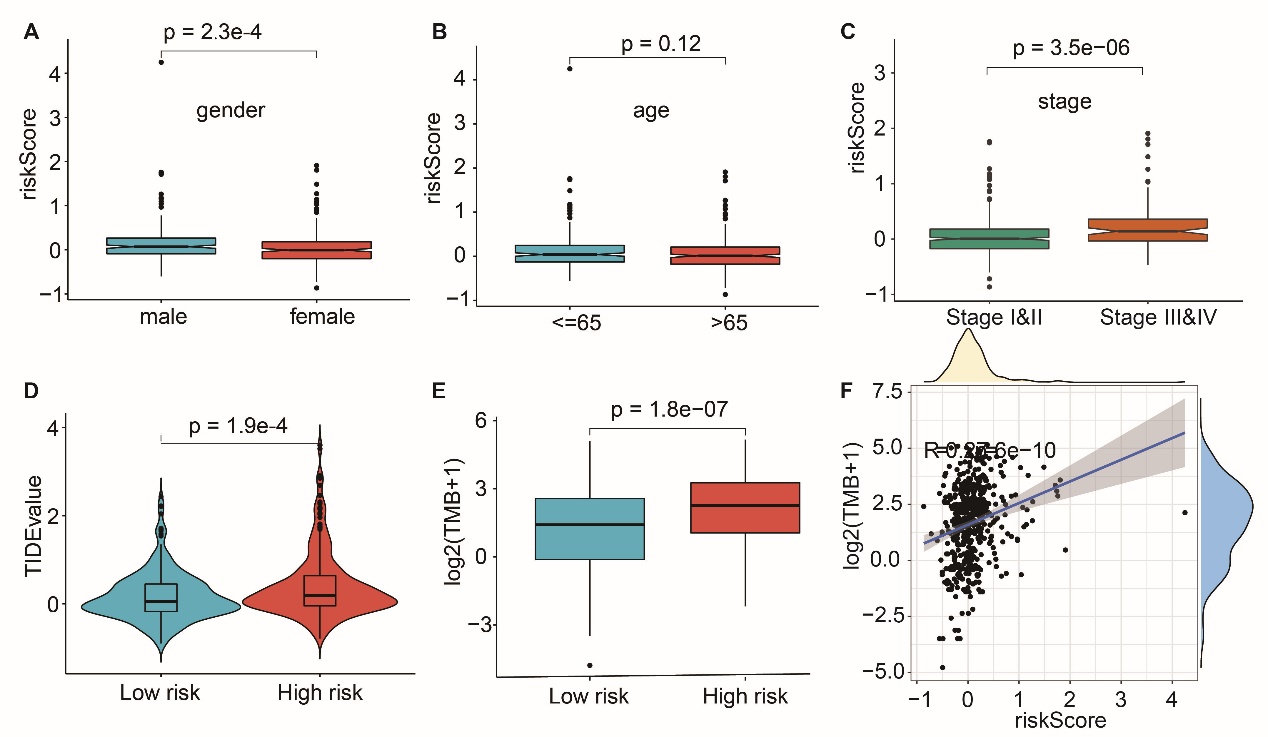


**Supplementary Figure 3. Correlation of the prognostic signature with clinicopathological features, tumor Immune dysfunction and exclusion (TIDE) and tumor mutation burden (TMB) in the TCGA dataset.** The differences in the risk score between groups are classified by gender (A), stage (B) and age (C). Comparisons of TIDE (D) and TMB (E) between the high- and LS groups. (F) Correlation analysis between TMB and risk.
